# Impact of concurrent parathyroid adenoma on the recurrence of differentiated thyroid carcinoma: a retrospective study

**DOI:** 10.55730/1300-0144.5947

**Published:** 2024-12-09

**Authors:** Derya ÇAYIR, Alev ÇINAR, Mehmet BOZKURT, Bahadır KÜLAH

**Affiliations:** 1 Department of Nuclear Medicine, University of Health Sciences, Ankara, Turkiye; 2 Department of Nuclear Medicine, Etlik City Hospital, Ankara, Turkiye; 3 Department of Nuclear Medicine, Memorial Health Group, İstanbul, Turkiye; 4 Department of General Surgery, A Life Park International Academic Hospital, Ankara, Turkiye

**Keywords:** Parathyroid adenoma, differentiated thyroid carcinoma, recurrence

## Abstract

**Background/aim:**

The impact of concurrent parathyroid adenoma in differentiated thyroid carcinoma remains unclear. This study compared the recurrence rates of patients operated on for differentiated thyroid carcinoma with concomitant parathyroid adenoma and those operated on for thyroid carcinoma without detected parathyroid adenoma.

**Materials and methods:**

This retrospective study examined 340 patients who underwent total thyroidectomy for thyroid carcinoma at our institution. We compared patients with concurrent parathyroid adenoma to those without, assessing clinical, laboratory, surgical, and histopathological findings and thyroid carcinoma recurrence over a 6-year postoperative period.

**Results:**

Among all patients, 53/340 (16%) were identified with parathyroid adenoma and were predominantly over the age of 50 years (p < 0.00). There was a significant difference between patients with and without parathyroid adenoma regarding the preoperative serum Ca level (11.21 ± 0.41 mg/dL versus 9.5 ± 0.36 mg/dL, respectively), preoperative serum P level (2.3 ± 0.3 mg/dL versus 3.7 ± 1.4 mg/dL, respectively), and preoperative serum PTH level (142 ± 89.3 pg/mL versus 53.7 ± 11.5 pg/mL, respectively) (p < 0.001 overall). Residual thyroid tissue was detected in 215/340 (63%) patients, and a significant difference existed between patients with parathyroid adenoma (20/53, 38%) and without it (195/287, 68%) (p < 0.00). No significant differences were observed in surgical and/or tumor characteristics between patients with and without parathyroid adenoma.

**Conclusion:**

Our findings suggest that parathyroid adenoma has no direct impact on the recurrence of differentiated thyroid carcinomas in the short term. However, our small cohort size limits the generalizability of these results, indicating a need for further studies with larger patient populations and longer follow-up durations.

## 1. Introduction

Differentiated thyroid cancer (DTC), comprising mainly papillary and follicular thyroid carcinomas, accounts for over 90% of all thyroid cancer cases and is the most prevalent form of thyroid malignancy. These cancers are characterized by their ability to maintain some degree of normal thyroid cell function, including iodine uptake and thyroglobulin (Tg) production. DTC generally has an excellent prognosis, with a 10-year survival rate exceeding 90% in most cases [1]. However, approximately 5–10% of patients develop distant metastases, which can significantly impact long-term outcomes [2]. The primary treatment for DTC is thyroidectomy, which may be total, near-total, or hemithyroidectomy, depending on the extent of the disease. Following surgery, radioactive iodine (RAI) therapy is often employed to ablate any remaining thyroid tissue and to treat microscopic disease [3]. Suppression of thyroid-stimulating hormones (TSH) with thyroid hormones is a standard postoperative strategy to reduce the risk of cancer recurrence. In cases where the cancer is refractory to RAI therapy or has advanced locally or metastasized, targeted therapies such as tyrosine kinase inhibitors like Sorafenib or Lenvatinib may be considered to improve patient outcomes [4]. The management of DTC has evolved significantly in recent years, moving toward a more personalized approach based on individual risk stratification and molecular profiling [5].

Primary hyperparathyroidism (PHPT) is a medical condition in which parathyroid glands produce excessive parathyroid hormones (PTH). The main causes of this condition are adenomas (in 80% of cases), multiglandular hyperplasia (in 15–20% of cases), and parathyroid carcinoma (in 1% of cases). If not treated, this slowly progressing disease can impact various body systems and lead to complications [6,7].

For patients with high calcium (Ca) levels, testing PTH levels is key to finding the cause and is one of the first tests effectuated. PHPT is diagnosed when there are high levels of both Ca and PTH in the blood and low phosphorus (P) levels. Initially, parathyroid ultrasound and parathyroid scintigraphy are recommended. These imaging techniques are used to locate the lesion in patients who have been biochemically confirmed to have hyperparathyroidism, not to diagnose the condition. Because parathyroid ultrasound and parathyroid scintigraphy are not very sensitive in diseases affecting multiple glands, like adenomas and hyperplasia, PTH measurement during surgery is used to avoid unsuccessful treatment. Surgery is the only way to cure the condition, and a parathyroidectomy is recommended for all patients with symptoms, especially those affecting the kidneys and bones [8].

This study aimed to compare the recurrence rate of patients operated on for thyroid carcinoma with concomitant parathyroid adenoma and those operated on for thyroid carcinoma without detected parathyroid adenoma.

## 2. Materials and methods

Ethical approval for this study was obtained from the University of Health Sciences Diskapi Yildirim Beyazit Training and Research Hospital’s Noninterventional Ethics Committee (No: 17.12.2018-57/26). We conducted a retrospective review of 340 patients who underwent total thyroidectomy at our hospital and were initially diagnosed with thyroid carcinoma from January 2014 to January 2019. Total thyroidectomy was preferred for 176 (52%) patients, and cervical lymph node dissection (CLND) was performed with total thyroidectomy on 164 (48%) patients. Lateral neck dissection was performed on 47 of the patients with CLND. Patients were excluded if they had nonpapillary thyroid carcinoma, such as follicular, medullary, or anaplastic carcinoma. Patients with concurrent PHPT and histologically confirmed parathyroid adenomas were classified as the hyperparathyroidism group. Those with secondary hyperparathyroidism were not included in the study. A nuclear medicine specialist collected the data on patients operated on by the same experienced surgeon.

We reviewed the imaging and laboratory data of the patients from our hospital’s records. Ultrasound findings, histopathological reports, and tumor size, as well as serum PTH, albumin corrected Ca, P, vitamin D (25-OH-Vit D), TSH, Tg, and anti-Tg antibody (TgAb) levels were examined retrospectively. Additionally, parathyroid scintigraphy and postoperative thyroid scintigraphy images obtained in the Nuclear Medicine Department were retrospectively assessed using a gamma camera. Two experienced nuclear medicine specialists performed the visual analysis of the scintigraphy images.

According to the latest guidelines of the American Thyroid Association, the patients were classified into low-risk, intermediate-risk, and high-risk groups. During 78.00 ± 15.49 months of follow-up or roughly 6 years, the patients were evaluated with biochemical and ultrasonography findings.

In this study, “recurrence” refers to the worsening or advancement of thyroid carcinoma after surgery, such as local relapse and/or metastasis observed over the 6-year follow-up period.

Initially, we calculated descriptive statistics for the variables, including means, medians, counts, and percentages. We employed Student’s t-test to compare numerical variables between the two groups. Furthermore, logistic regression analysis was performed to explore risk factors associated with parathyroid adenoma. SPSS 17 (SPSS Inc., Chicago, IL, USA) was used to evaluate the results. Values of p < 0.05 were considered significant.

## 3. Results

Of the 340 patients, 43 (13%) were male, and 297 (87%) were female. The mean age was 50 ± 12.6 (range: 38–62) years. According to the postoperative pathology results of the patients, parathyroid adenoma was detected in 53 (16%) individuals. Nine (17%) parathyroid adenomas were incidentally identified, while 44 (83%) were detected preoperatively through scintigraphy. The mean parathyroid adenoma size was found to be 14.3 ± 7.1 mm. Demographic characteristics of all patients are detailed in Table 1.

The mean preoperative serum Ca level was 11.21 ± 0.41 mg/dL, the mean preoperative serum P level was 2.3 ± 0.3 mg/dL, and the mean preoperative serum PTH level was 142 ± 89.3 pg/mL in the patients with adenoma. The mean preoperative serum Ca level was 9.5 ± 0.36 mg/dL, the mean preoperative serum P level was 3.7 ± 1.4 mg/dL, and the mean preoperative serum PTH level was 53.7 ± 11.5 pg/mL in the patients without adenoma. There was a significant difference between patients with and without parathyroid adenoma regarding serum preoperative Ca, P, and PTH levels (p < 0.00).

The mean age of those with parathyroid adenoma was 59 ± 12 years, and only 6/53 (11%) of these patients were male. Total thyroidectomy was performed for 22/53 (41.5%) of these patients, and CLND was added to the procedure for 31/53 (58.5%) patients with parathyroid adenoma. The mean postoperative TSH level was 44.19 ± 12.66 mIU/L, the mean postoperative Tg level was 1.1 (0.2–3.3) ng/mL, and the mean postoperative TgAb level was 10.45 ± 6.43 IU/mL in patients with parathyroid adenoma. The mean preoperative urine calcium level in this group was 312 ± 85 mg/day.

The mean age of the group without parathyroid adenoma was 48 ± 12 years, and 37/287 (13%) patients in this group were male. Total thyroidectomy was performed for 154/287 (54%) of these patients, and CLND was added for 133/287 (46%) patients without parathyroid adenoma. The mean postoperative TSH level was 46.13 ± 25.28 mIU/L, the mean postoperative Tg level was 1.1 (0.2–3.8) ng/mL, and the mean postoperative TgAb level was 11.15 ± 8.61 IU/mL in individuals without parathyroid adenoma. No significant differences were observed in the postoperative serum Tg, TSH, and TgAb levels between patients with and without parathyroid adenoma.

Residual tissue was detected in 215 (63%) patients. There was a significant difference between patients with parathyroid adenoma (20/53, 38%) and those without parathyroid adenoma (195/287, 68%) regarding the presence of residual thyroid tissue (p < 0.00).

The pathology results revealed papillary microcarcinoma (mPTC) in 195/340 (57%) patients, with a mean tumor size of 9.0 (4.0–14.0) mm in the entire group. Multifocality was present in 152 (45%), lymph node metastasis (LNM) in 30/340 (18%), capsule invasion in 43 (13%), and extrathyroidal extension (ETE) in 17/340 (5%) patients. Demographic, clinical, laboratory, and scintigraphic findings and surgical and tumor characteristics of the patients with and without parathyroid adenoma are detailed in Table 2.

In the group with parathyroid adenoma, 33/53 (62%) patients had mPTC, and 20/53 (38%) had papillary thyroid carcinoma (PTC). The mean tumor size was 6.0 (3.0–13.0) mm. Multifocality was present in 26 (49%), LNM in 5 (16%), capsule invasion in 4 (8%), and ETE in 2 (4%) patients.

The risk groups were classified as low (180/340, 53%), intermediate (126/340, 37%), or high (34/340, 10%) risk. High-dose RAI (100–150 mCi) was given to 37/340 (11%) patients, and low-dose RAI (30 mCi) was administered to 157/340 (46%) patients for ablation/therapy.

In the group without parathyroid adenoma, the tumor size was 9.0 (4.0–15.0) mm. Multifocality was present in 126 (44%), LNM in 25 (19%), capsule invasion in 39 (14%), and ETE in 15 (5%) of these patients. No significant differences were observed in surgical and/or tumor characteristics between patients with and without parathyroid adenoma.

The classic papillary variant was observed in 144/340 (42%) patients, while 95/340 (28%) had the follicular variant, 71/340 (21%) had other variants, and 30/340 (9%) had other types (e.g., tall cell variant, diffuse sclerosing variant, or hobnail variant). There were three cases of permanent hypoparathyroidism following surgery. These patients experienced this condition due to complications from the total thyroidectomy procedure, which can occur due to inadvertent damage or removal of the parathyroid glands during surgery.

Our findings suggest that the likelihood of concurrent parathyroid adenoma in patients with thyroid carcinoma increases 6-fold in individuals over the age of 50 (p < 0.00).

## 4 Discussion

The simultaneous occurrence of thyroid and parathyroid disorders was first demonstrated in 1947 [9]. While the combination of medullary thyroid carcinoma and PHPT is frequently observed in multiple endocrine neoplasia type 2A (MEN-2A), parathyroid adenomas alongside nonmedullary thyroid carcinomas are relatively uncommon [10–12]. Katz and Kong [13] conducted a study identifying preclinical PHPT in 36 of 800 patients undergoing surgery for thyroid diseases. Among the 36 patients with preclinical PHPT, nine were found to have nonmedullary thyroid carcinoma.

Radiation to the head and neck region and a positive family history are risk factors for both thyroid carcinoma and parathyroid adenoma. However, the exact nature of this relationship remains inconclusive. While some researchers consider it coincidental, others have suggested that it may be associated with increased endogenous Ca levels or growth factors such as epithelial growth and insulin-like growth factors [14].

In patients diagnosed with PTC, parathyroid adenomas can sometimes resemble metastatic lymph nodes during the preoperative evaluation. The concurrent presence of hyperparathyroidism in thyroid disease is frequent. Therefore, relying solely on serum Ca and PTH levels may not provide a comprehensive assessment in such cases. Computerized tomography can also be a valuable tool in identifying slowly progressing asymptomatic hyperparathyroidism. Additionally, Tc-99m MIBI scintigraphy is the most sensitive diagnostic method for localizing adenomas today; another advantage of this method is that it can identify ectopic tissues. The recommended method for diagnosing thyroid pathologies accompanying PHPT combines scintigraphy, ultrasound, and ultrasound-guided fine-needle aspiration biopsy. Although coexisting thyroid carcinoma and parathyroid adenoma are rare, this association should be considered in patients with PHPT to prevent repeated surgery.

PTC is the most common type of thyroid carcinoma, accounting for approximately 85% of differentiated thyroid carcinomas. It is generally more common in women between 30 and 40, with a female-to-male ratio of 2–3 to 1 [15–17]. Surgical treatment is applied in treating PTC. RAI therapy can be used to ablate residual thyroid tissue or to treat metastases. In addition to the recurrent laryngeal nerve, another significant consideration in thyroid surgery is preserving the parathyroid glands. After removing the thyroid gland, a thorough examination is conducted to determine if the parathyroid glands remain intact. Hypoparathyroidism is one of the most common complications related to thyroid surgery. After subtotal thyroidectomy, there is less hypoparathyroidism than total thyroidectomy. It can be temporary or permanent and usually manifests itself with hypocalcemia. Total thyroidectomy can be performed while preserving the parathyroid glands. The parathyroid hormone is a crucial regulator of Ca metabolism, promoting the release of Ca from bone and its retention in the gastrointestinal system. Symptoms of hypocalcemia due to damage to the glands generally appear around the third day after surgery. In our study, the mean postoperative Ca level was 9.4 ± 0.6, and the mean postoperative PTH level was 51 ± 21.1 in all patients.

PTC generally has an excellent prognosis. Even when the disease recurs, the prognosis remains favorable, which has sparked discussion regarding the optimal approach to PTC surgery. Effective surgical intervention must achieve a delicate balance between minimizing potential morbidities and ensuring prolonged survival. Independent risk factors for long-term survival include the patient’s age, ETE, and the presence of distant metastasis. LNM is not considered an independent risk factor. Age is a crucial prognostic factor, with more aggressive courses observed in those over 60 and under 20. Many studies have shown a relationship between age and disease mortality. The limit is 40 years old for male and 50 for female patients [18]. However, independent risk factors for recurrence-free survival include being male and the presence of LNM and ETE.

In this study, total thyroidectomy and CLND were performed on 31 (58.5%) patients with parathyroid adenoma, and 133 (46%) patients without parathyroid adenoma underwent total thyroidectomy and CLND. However, during follow-up, no disease recurrence was observed in either group when patients were treated with TSH-suppressive therapy. In a cohort study of 885 patients with PTC, it was observed that patients with tumors smaller than 1.5 cm had no recorded deaths attributed to PTC. For patients presenting with larger tumors without ETE, the mortality rate was 16%.Meanwhile, patients with larger tumors that exhibited ETE had a higher mortality rate of 3% [19]. Multifocality in PTC is a frequent finding, occurring in approximately 20–30% of all cases, and this rate can be as high as 75% in cases associated with radiation exposure [20]. Although the presence of multiple tumor foci has not been directly associated with increased mortality, it is noteworthy that patients with multifocal PTC are twice as likely to develop LNM and three times more likely to develop distant metastases compared to those with unifocal disease [21]. In our study, although the presence of parathyroid adenoma was slightly higher in the group with multifocality (49%) than in the one without adenoma (44%), no statistically significant difference was found in recurrence-free survival (p = 0.488) during the 6-year follow-up period.

Tumor size is a critical factor to consider in carcinoma, arguably the most crucial, and it serves as the primary parameter in all classification systems. A study by Machens et al. [22] showed that PTC is less likely to metastasize until the tumor size surpasses 20 mm. In classifying risk groups, a threshold of 4 cm is commonly used, classifying patients with tumors larger than 4 cm as being of high risk, with that risk increasing with age. In our study, the maximum tumor size was 21 mm. No statistically significant difference was found in recurrence-free survival between the group with parathyroid adenoma and those without in the 6-year follow-up period (p = 0.154).

PTC can invade the thyroid capsule and extend beyond the thyroid itself. This pattern of ETE categorizes the disease as high-risk. Malignant cases often exhibit invasion of lymphatic or blood vessels upon histological examination. In assessments of recurrent PTC cases, a significant correlation has been established between lymphovascular invasion and recurrence, and the presence of multifocality in PTC has been a recognized feature for quite some time. A previous study reported observing this feature in 20% of cases; when sections were reduced, the authors found that the rate increased to 85% [23]. Our study showed no statistically significant difference between the groups regarding capsular invasion or ETE (p = 0.322 and p = 1.00, respectively).

Regional LNM can develop in 20–90% of PTC cases [24]. While some authors have argued that LNM does not impact the prognosis of PTC, a study analyzing a database of 9904 patients found that LNM was linked to poorer prognosis [25]. Our study showed no statistically significant difference between the groups regarding ETE (p = 0.93).

In conclusion, prognostic evaluations for PTC currently rely heavily on histological analyses conducted after surgery. Our study, which included 340 patients, did not reveal a statistically significant correlation between pathological findings and the occurrence of parathyroid adenoma. Additionally, throughout the initial 6 years of postoperative monitoring, none of the patients exhibited alterations in serum Ca levels or advancement of thyroid carcinoma. The study’s small sample size limited our ability to thoroughly investigate the influence of parathyroid adenoma on the recurrence of differentiated thyroid carcinomas. However, among the subgroup of 53 patients with parathyroid adenoma, it was notable that all were over 50 years old. More research should be conducted on the possible prognostic significance of parathyroid adenoma in patients with differentiated thyroid carcinoma, especially with longer follow-up periods and larger cohorts.

## Figures and Tables

**Table 1:** Demographic characteristics of the 340 patients.

Patient characteristics	N (%)

Age ± SD (years) (range)	50 ± 12.6

Sex	
Female	297 (87%)
Male	43 (13%)

Presence of parathyroid adenoma	53 (16%)

Mode of diagnosis	
Incidental	9 (17%)
Nonincidental	44 (83%)

Average adenoma size ± SD (mm) (range)	14.3 ± 7.1

Preoperative albumin corrected serum Ca (mg/dL)	9.8 ± 0.7

Preoperative serum P (mg/dL)	3.3 ± 0.9

Preoperative serum PTH (pg/mL)	74 ± 12.2

25-OH-Vit D (μg/L)	28.2

Postoperative albumin corrected serum Ca (mg/dL)	8.8 ± 2.1

Postoperative serum P (mg/dL)	3.5 ± 0.7

Postoperative serum PTH (pg/mL)	49.3 ± 11.5

Presence of residual thyroid tissue	215 (63%)

Tumor type	
mPTC	195 (57%)
PTC	145 (43%)

Mean tumor size (mm) (range)	9.0 (4.0–14.0)

Tumor characteristics	
Multifocality	152 (45%)
Capsular invasion	43 (13%)
ETE	17 (5%)
LNM	30 (18%)

* Papillary thyroid microcarcinoma (mPTC); papillary thyroid carcinoma (PTC); extrathyroidal extension (ETC); lymph node metastasis (LNM).

**Table 2:** Demographic features of patients with and without parathyroid adenoma and p-values.

Patient characteristics	With parathyroid adenoma (53/340)	Without parathyroid adenoma (287/340)	p-values

Age ± SD (years)	59 ± 12	48 ± 12	0.105

Sex			
Female	47 (89%)	250 (87%)	0.752
Male	6 (11%)	37 (13%)	

Surgery type			
Total thyroidectomy	22 (41.5%)	154 (54%)	0.104
Total thyroidectomy + CLND	31 (58.5%)	133 (46%)	

Preoperative albumin corrected Ca (mg/dL)	11.21 ± 0.41	9.5 ± 0.36	<0.001

Preoperative serum P (mg/dL)	2.3 ± 0.3	3.7 ± 1.4	<0.001

Preoperative serum PTH (pg/mL)	142.9 ± 89.3	53.7 ± 11.5	<0.001

25-OH-Vit D (μg/L)	27	28.5	0.738

Postoperative serum Ca (mg/dL)	9.1 ± 0.8	8.7 ± 1.1	0.625

Postoperative serum P (mg/dL)	3.1 ± 0.2	3.7 ± 0.4	0.431

Postoperative serum PTH (pg/mL)	51.4 ± 17.3	48.8 ± 13.8	0.764

Postoperative Tg (ng/mL)	1.1 (0.2–3.3)	1.1 (0.2–3.8)	0.852

Postoperative TSH (mIU/L)	44.19 ± 12.66	46.13 ± 25.28	0.593

Postoperative TgAb (IU/mL)	10.45 ± 6.43	11.15 ± 8.61	

Presence of residual thyroid tissue	20 (38%)	195 (68%)	<0.001

Tumor type			
mPTC	33 (62%)	162 (56%)	0.431
PTC	20 (38%)	125 (44%)	

Mean tumor size (mm) (25–75%)	6.0 (3.0–13.0)	9.0 (4.0–15.0)	0.092

Tumor characteristics			
MultifocalityM	26 (49%)	126 (44%)	0.488
Capsular invasion	4 (8%)	39 (14%)	0.322
ETE	2 (4%)	15 (5%)	1.00
LN	5 (16%)	25 (19%)	0.93

* Cervical lymph node dissection (CLND); papillary thyroid microcarcinoma (mPTC); papillary thyroid carcinoma (PTC); extrathyroidal extension (ETE); lymph node metastasis (LNM).
